# Data on multiple body parameters, microclimatic variables, and subjective assessment of thermal sensation monitored in outdoor environment

**DOI:** 10.1016/j.dib.2017.03.045

**Published:** 2017-04-08

**Authors:** Katerina Pantavou, Anastasios Mavrakis, Georgios K. Nikolopoulos

**Affiliations:** a Department of Environmental Physics and Meteorology, Faculty of Physics, National & Kapodistrian University of Athens, Athens, Greece; b Department of Economic and Regional Development, Panteion Panepestimion Ikonomikon kai Politicon Epistimon, Athens, Greece; c Medical School, University of Cyprus, Nicosia, Cyprus

**Keywords:** Field surveys, Questionnaire, Skin temperature, Thermal sensation

## Abstract

This paper describes two sets of data on multiple body parameters of five participants, on microclimatic variables, and on self-reported assessment of thermal responses, all monitored in the same outdoor urban environment. Data were collected during three seasons, summer, autumn and winter 2010–2011, in the city of Athens, Greece. Part of these data, collected during the summer period, is related to the research article entitled "Case study of skin temperature and thermal perception in a hot outdoor environment." (Pantavou et al., 2014) [1].

**Specifications Table**

**Table:** 

Subject area	*Biometeorology*
More specific subject area	*Outdoor thermal sensation*
Type of data	*Excel files*
How data was acquired	*Data were collected during field surveys that involved measurements of body parameters and microclimatic monitoring along with a subjective assessment of thermal sensation based on questionnaires.*
Data format	*Raw, analyzed*
Experimental factors	*The participants were five volunteers, 3 males and 2 females, aged between 24 and 46 years old. They were wearing clothing of their choice and were mainly standing while undergoing light activity.*
Experimental features	*The measurements were taken in three seasons: summer, autumn, and winter 2010–2011.*
Data source location	*Athens (37*^*°*^*59′20″N, 23*^*°*^*43′41″E), Greece.*
Data accessibility	*Data is with this article.*

**Value of the data**

• The data can be used to examine the thermo-physiological responses of human body to meteorological variables.
• The datasets can be used to investigate the potential association of thermo-physiological responses of the human body to subjective thermal sensation and to explore potential differences between individuals.
• Meteorological data allow the estimation of thermal indices that can be compared to the thermal sensation reported by the participants.

## 1 Data

The present article contains data on body parameters, microclimatic variables, and subjective assessment of thermal sensation, overall comfort and preference regarding thermal sensation, reported through questionnaires answered by five individuals. The datasets are in two Excel files: BodyParametersData.xlsx and QuestionnaireData.xlsx. The BodyParametersData.xlsx contains in different sheets per minute measurements of body parameters for each participant. The QuestionnaireData.xlsx file contains data on self-reported thermal responses based on a questionnaire and on meteorological variables monitored during the completion of the questionnaire.

## 2 Experimental design, materials and methods

### 2.1 General framework

The data were collected during field questionnaire-based surveys investigating the thermal sensation of a Mediterranean population [2]. Overall, five individuals (Table 1) volunteered to wear the multi-sensor device SenseWear Pro II Armband (BodyMedia Pittsburgh, PA) [1] and to self-report, based on the questionnaire, their subjective responses regarding thermal sensation, overall comfort, and preference [2]. The surveys were conducted in July, October and February 2010–2011. The time frame varied by season [2]. Measurements were taken at three different sites in Athens, Greece; Syntagma Square, Ermou Street, and Flisvos coast.

### 2.2 Monitoring of body parameters

The participants (Table 1) were healthy, acclimatized, and simultaneously exposed to the same outdoor thermal environment during the whole period of the experiments. They were wearing the clothing of their choice, and were mainly standing while undergoing light activity corresponding to a metabolic rate of 93 W m^−2^ [3]. The participants worn a multi-sensor device SenseWear Pro II Armband (BodyMedia Pittsburgh, PA) on the triceps of their right arm [1]. The SenseWear collected data on skin temperature, ambient air temperature, heat flow, galvanic skin response, 2-axis acceleration and metabolic rate in participants׳ arm at 1-min interval. The data are included in the BodyParametersData.xlsx. All participants were involved in the summer field surveys (Table 2).

### 2.3 Monitoring of microclimatic conditions

A mobile meteorological station was used to monitor microclimatic conditions near the participants at the height of 1.1 m above the ground [2]. The monitored meteorological variables were: ambient air temperature (T_air_), relative humidity (RH), average and maximum wind speed (WS, WS_max_), globe temperature (T_globe_), total (SR_↓_) and reflected (SR_↑_) solar radiation, and total radiation (TR_↓_, TR_↑_) on a horizontal plane, at 1-min interval. The ground surface temperature (T_ground_) was measured at the moment and point the participants were completing the questionnaire.

Total (SR_↓_), reflected (SR_↑_) solar radiation and total radiation (TR_↓_, TR_↑_) were used to estimate long wavelength radiation (IR_↓_, IR_↓_) per minute.

Three-minute average of all meteorological values were calculated and matched to the time the completion of the questionnaire started. Three-minutes was the required time for completing the questionnaire.

The three-minute average of the meteorological variables that correspond to each filled questionnaire are given in the QuestionnaireData.xlsx.

### 2.4 Subjective assessment of thermal responses

During the surveys, each participant was completing a questionnaire every 30 min. The participants were asked to fill in the questionnaire with direct reference to the specific moment and report their thermal sensation according to the symmetrical 7-degree dipolar scale −3, cold; −2, cool; −1, slightly cool; 0, neutral; +1, slightly warm; +2, warm; +3, hot [4]. Additional questions asked for the assessment of overall sensation (How do you feel overall at this place?) according to a 5-degree dipolar scale (−2, very comfortable; −1, comfortable; 0, neutral; +1, uncomfortable; +2, very uncomfortable) and for the participants׳ opinion about the weather on the experimental day (What is your opinion about the weather today?) in relation to air temperature (−1, cold; 0, neutral; +1, warm), humidity (−1, dry; 0, neutral; +1, humid) and wind (−1, calm; 0, neutral; +1, windy), for their preference related to overall sensation (How would you prefer to feel at this moment?; −1, cooler; 0, no change; +1, warmer) and the way it would improve (How do you think your thermal condition would improve at this moment?) in terms of air temperature, humidity, wind, irradiation according to a 3-degree dipolar scale (−1, lower; 0, no change; +1, higher). Moreover, the participants were asked to indicate their clothing ensembles in order to estimate clothing insulation (I_cl_, in clo) from the description of clothes according to ISO 9920 [5]. The data are included in the QuestionnaireData.xlsx.

## Funding sources

This research did not receive any specific grant from funding agencies in the public, commercial, or not-for-profit sectors.

## Figures and Tables

**Table 1:** Participants׳ characteristics.

Participants	Body Mass Index (BMI)	Handedness	Smoker
P1	20.90	Right Handed	Smoker
P2	27.68	Right Handed	Non-Smoker
P3	17.30	Right Handed	Non-Smoker
P4	22.99	Right Handed	Non-Smoker
P5	27.16	Left Handed	Non-Smoker

**Table 2:** Participants in the field surveys.

Summer		Autumn		Winter	
Date	Participants	Date	Participants	Date	Participants
15/07/2010	P1, P2, P3, P4, P5	16/10/2010	P1, P2, P3, P4	09/02/2011	P1, P2
16/07/2010	P1, P2, P3, P4, P5	17/10/2010	P1, P2, P3, P4	12/02/2011	P1, P2
17/07/2010	P1, P2, P3, P4, P5	20/10/2010	P2, P3, P4	13/02/2011	P1, P2
18/07/2010	P1, P2, P3, P4, P5	23/10/2010	P2, P3, P4, P5	26/02/2011	P1, P2
20/07/2010	P1, P2, P3, P4, P5			27/02/2011	P1, P2
21/07/2010	P1, P2, P3, P4, P5				
